# Thermal and Osmotic Tolerance of ‘Irukandji’ Polyps: Cubozoa; *Carukia barnesi*

**DOI:** 10.1371/journal.pone.0159380

**Published:** 2016-07-21

**Authors:** Robert Courtney, Sally Browning, Tobin Northfield, Jamie Seymour

**Affiliations:** 1 Australian Institute for Tropical Health and Medicine, Division of Tropical Health & Medicine, James Cook University, Cairns, Queensland, Australia; 2 Centre for Tropical Environmental and Sustainability Science, College of Science and Engineering, James Cook University, Cairns, Queensland, Australia; UC Irvine, UNITED STATES

## Abstract

This research explores the thermal and osmotic tolerance of the polyp stage of the Irukandji jellyfish *Carukia barnesi*, which provides new insights into potential polyp habitat suitability. The research also targets temperature, salinity, feeding frequency, and combinations thereof, as cues for synchronous medusae production. Primary findings revealed 100% survivorship in osmotic treatments between 19 and 46‰, with the highest proliferation at 26‰. As salinity levels of 26‰ do not occur within the waters of the Great Barrier Reef or Coral Sea, we conclude that the polyp stage of *C*. *barnesi* is probably found in estuarine environments, where these lower salinity conditions commonly occur, in comparison to the medusa stage, which is oceanic. Population stability was achieved at temperatures between 18 and 31°C, with an optimum temperature of 22.9°C. We surmise that *C*. *barnesi* polyps may be restricted to warmer estuarine areas where water temperatures do not drop below 18°C. Asexual reproduction was also positively correlated with feeding frequency. Temperature, salinity, feeding frequency, and combinations thereof did not induce medusae production, suggesting that this species may use a different cue, possibly photoperiod, to initiate medusae production.

## Introduction

Tropical Australian cubozoans are highly seasonal, with the medusa stage usually arriving along the tropical coastlines during the monsoonal summer months [1–7]. This ‘stinger season’ in Australia is typically between November and May, which has been reported to begin earlier, and last longer, in warmer areas closer to the equator such as in the Gulf of Carpentaria [3,8,9]. At least part of the observed seasonality of the medusa stage is thought to be driven by the complex life cycle of cubozoans.

Cubozoans have a metagenic life cycle that alternates between benthic polyps, which reproduce asexually, and pelagic medusae, that reproduce sexually [2,4,10–16]. Medusa production in cubozoans is variable and can be in the form of complete metamorphosis of the polyp into a medusa [11,12,16–20], metamorphosis that leaves behind a small amount of regenerative material which is able to develop into a polyp (residuum) [21], and monodisc strobilation that leaves behind a polyp able to continue asexual reproduction of more polyps [22,23]. Some cubozoan polyps are known to use environmental factors, such as temperature [19,24,25], a combination of temperature and light intensity [18], photoperiod [3], or reduced food availability [26], as a cue to induce synchronous medusa production. It is thought that once medusa production is initiated, it can be punctuated by significant rainfall events, for example *Chiropsella bronzie*, or is continuous, as in *Chironex fleckeri* [2,3]. Therefore, medusae production by the polyps is considered to be one factor that drives the seasonal fluctuation in abundance of the medusae stage [2–4,20].

The medusa stage of *Carukia barnesi*, the Irukandji jellyfish, often causes painful and potentially fatal stings [1,6,7,27,28]. A sting from *C*. *barnesi* is also renowned for causing Irukandji syndrome which often requires hospitalization for treatment [1,27,28]. Considerable costs are associated with treating sting victims and lost revenue to the tourism industry annually is believed to be substantial [1]. Although only the medusa stage of this cubozoan poses a threat to humans, the polyp stage is expected to contribute to the seasonal periodicity through the synchronous timing of medusae production causing the start of the stinger season. Similarly, the abundance of medusae present during the season may be reflective of the success of the polyp stage.

The polyp stage also contributes to the abundance of medusae present by asexually reproducing more polyps, thus increasing the number of polyps able to produce medusae at any one time. Thermal, osmotic and food availability parameters are known to influence the rate of asexual reproduction of the polyp stage of *Alatina* nr *mordens* [26,29] and *Carybdea marsupialis* [30–32]. However, in most instances, the *in situ* parameters of the polyps are not known due to the unknown location of the polyps, and to date, cubozoan polyps have only been discovered *in situ* for two species: *C*. *fleckeri* and *C*. *marsupialis* [4,12,20,32]. Because of this, laboratory-based cultures of cubozoan polyps are essential for exploring the factors that affect polyp survivorship and proliferation in order to deduce where the polyps may reside, which may also shed light on the factors affecting the seasonal influx of medusae.

*Carukia barnesi*, like other Australian cubozoans, exhibits a marked seasonality. Little is known about the ecology of the early life stages of this species [22], even though it was discovered as causing Irukandji syndrome over 50 years ago [6,33,34]. The medusa stage of *C*. *barnesi* is considered oceanic and is typically found around coral reefs and islands and under certain condition along beaches [1,5,6,34,35]. However, nothing is known about the thermal and osmotic preference or the location of the polyp stage *in situ*. Therefore, a series of temperature, salinity, and feeding frequency experiments were conducted on the polyp stage in order to gain a better understanding of the ecology of this species.

The primary aims of this research were: 1) to determine the thermal and osmotic tolerance of the polyps, and use these data to discuss where these polyps may live; 2) to explore the effects of feeding frequency on survivorship and asexual reproduction and discuss intrinsic population growth rates under different feeding regimes; 3) to determine the effect of thermal and osmotic parameters, and combinations thereof, as well as feeding frequency, as cues for medusae production.

## Method

### Specimen Identification

*Carukia barnesi* is a small species in the family Carukiidae. The medusa stage has a bell height of up to 35 mm and tentacles up to 1.2 m long [6,34–37] with a single tentacle that extends from each of the four pedalia; rhopaliar niches with horns; and tentacles that have an alternating pattern of large and small nematocyst crescents that resemble “neckerchiefs” [6,34–36]. The polyps of *C*. *barnesi* average 0.9 mm in length and have 11 capitate tentacles on average. The polyps asexually reproduce ciliated swimming polyps and produce medusae through monodisc strobilation (see [22]). Little is known about the distribution of this species; however, medusae are often present along the north-eastern coast of Australia during the warm monsoonal months (November to May) between Lizard Island and Fraser Island [5,6,34–36].

### Experimental Design and Pre-treatment

This project consisted of two separate experiments in which polyps of *C*. *barnesi* were exposed to different temperature, salinity and/or feeding frequency treatments that were monitored for survivorship, population increase through asexual reproduction, and medusae production following a previously published method [26]. The *C*. *barnesi* polyps were derived from the laboratory-based culture outlined in Courtney et al. [22], which was established approximately six months prior to these experiments from wild caught adult medusae. This polyp culture was maintained at a constant temperature of 28°C ± 0.5°C and a salinity level of 33‰ ± 0.5‰.

Both of the experiments presented here were conducted in 24 well sterile micro-plates and only 12 of the wells within each plate were used to allow for blank wells between different treatments. Each well had a surface area of 11.5 cm^2^ and a volume of 3.5 ml. The mature polyps of *C*. *barnesi* produce swimming stage polyps through lateral budding [22]. These swimming polyps were harvested from the primary cultures and transferred into the 24 well micro-plates at a density of approximately 15 swimming polyps per well. As these polyps settled and developed into secondary polyps, they were fed freshly hatched *Artemia* sp. nauplii to satiation (approximately 20 *Artemia* sp.) every seven days. A complete water exchange was carried out 24 hours post each feeding event with filtered artificial seawater. The water quality parameters were maintained at the original culture parameters (28°C ± 0.5°C and 33‰ ± 0.5‰) for four weeks prior to any experimental trials to allow the polyps to acclimate, grow to maturity, and begin asexual reproduction. During this time, and during all experiments, a photoperiod of 13 hours light: 11 hours dark was maintained. These polyps were then exposed to one of the conditions outlined below; polyps were only exposed to one treatment, there was no mixing of water between replicates or treatments, and all treatments in each experiment were conducted simultaneously.

The first experiment consisted of 80 thermal and osmotic treatments (see Method: Thermal and Osmotic Effects on Survivorship and Asexual Reproduction), which were monitored using a binocular dissection microscope, for polyp survival, asexual reproduction, and medusae production over a six-week period. Each treatment comprised six independent replicates and each replicate consisted of an average of 18.63 polyps at the beginning of the experiment (i.e., $\bar{x}$ = 18.63, *SD* = 5.87 polyps; housed in each of 480 wells). Each replicate (well) was fed approximately 20 *Artemia* nauplii once per week and this quantity was increased as the polyp numbers increased (i.e., approximately one *Artemia* per polyp per week). Each feeding event was followed by a complete water exchange with artificial seawater of the same parameters as each experimental treatment (temperature and salinity).

The second experiment required a further five treatments to determine the effects of feeding frequency on asexual reproduction, survivorship, and medusae production (see Method: The Effects of Feeding Frequency on Survivorship and Asexual Reproduction). This experiment was conducted at the original polyp culture conditions of 28°C ± 0.5°C and 33‰ ± 0.5‰ over a six-week period. Each treatment comprised six replicates and each replicate consisted of an average of 20.5 polyps at the beginning of the experiment (i.e., $\bar{x}$ = 20.50, *SD* = 2.79 polyps; housed in each of 30 wells). Each replicate (well) was fed approximately 20 *Artemia* sp. nauplii per feeding event and this quantity was increased as the polyp numbers increased (i.e., one *Artemia* per polyp per feeding event).

### Thermal and Osmotic Effects on Survivorship and Asexual Reproduction

To determine the thermal and osmotic tolerance of *C*. *barnesi* polyps, and the effects these treatments had on survival, asexual reproduction and medusae production, replicates of polyps (as described above) were exposed to an 80 combination matrix of eight temperatures (11, 14, 18, 21, 25, 28, 31, and 34°C ± 0.5°C) and ten salinities (16, 19, 22.5, 26, 29, 33, 36, 39, 42.5, and 46‰ ± 0.5‰) over a period of six weeks. Each incubation temperature was maintained by partially submerging each test plate into one of eight temperature controlled water baths for the duration of the experiment. The range of temperatures selected encompasses the potential thermal regime a polyp may experience in the Coral Sea (temperatures commonly below 11°C at depths of approximately 500 m) and along the adjacent Australian coastline including estuarine environments (temperatures commonly occur above 34°C in shallow estuarine pools). The salinity range tested encompasses common salinities found in the Coral Sea and also included both hypersaline and hyposaline conditions that are commonly found in estuarine environments. These environmental parameters were explicitly targeted to deduce the thermal and osmotic tolerance of the polyp stage. Prior to the beginning of this experiment each replicate was photographed and the number of polyps in each well was determined. Every seven days during the six-week experiment, each replicate was fed and water exchanged (as described above). Each replicate was again photographed and the number of polyps in each well was determined in seven-day intervals during the six-week experiment.

To determine the thermal and osmotic tolerance of polyps, the change in population numbers was determined in seven-day intervals during the six-week experiment. To allow for comparisons between treatments, a model was fitted across all treatments that described final polyp density as a function of initial density, temperature, and salinity. We assumed that the final polyp density was proportional to the initial density, functions of temperature *F*(*T*) and salinity *G*(*S*), and a parameter *a* which describes the maximum population change at optimal conditions such that,

$$P_{final}\;\;=\;\;aF\left(T\right)G\left(S\right)P_{initial}.$$ (1)

Thus, we assume independent effects of temperature and salinity on growth rates. Preliminary analyses suggested including interactive effects between temperature and salinity did not qualitatively change the results. We model *F*(*T*) and *G*(*S*) as asymmetrical modified Gaussian functions centered around the optimal temperature and salinity, respectively such that,

$$F\left(T\right)=\;\{\begin{array}{c} \text{exp}\left[\frac{-{\left(T_{opt}-T\right)}^{2}}{\sigma_{t,l}{}^{2}}\right],\;for\;T<T_{opt}\\ \text{exp}\left[\frac{-{\left(T_{opt}-T\right)}^{2}}{\sigma_{t,h}{}^{2}}\right],\;for\;T>T_{opt} \end{array}$$ (2)

$$G\left(S\right)=\;\{\begin{array}{c} \text{exp}\left[\frac{-{\left(S_{opt}-S\right)}^{2}}{\sigma_{s,l}{}^{2}}\right],\;for\;S<S_{opt}\\ \text{exp}\left[\frac{-{\left(S_{opt}-S\right)}^{2}}{\sigma_{s,h}{}^{2}}\right],\;for\;S>S_{opt} \end{array},$$ (3)

where *T*_*opt*_ and *S*_*opt*_ describe the optimal temperature and salinity, respectively. The σ_*i*,*j*_^2^ parameters represent the slope for independent variable *i* (temperature or salinity) and *j*, the side of the curve (low, or high). We assume that the final polyp density follows a negative binomial distribution, and fit the models and calculated the log-likelihood, maximum likelihood estimates and 95% maximum likelihood profile confidence limits using the bbmle package, version 1.0.18 [38], in the statistical package R, version 3.2.4 [39,40]. The raw data file and R code has been included in Supporting Information (S1 Dataset and S1 R Code). To evaluate the effects of salinity and temperature, we used AIC values (Akaike Information Criterion) to compare the full model with models containing only temperature or salinity (i.e., *P*_*final*_ = *aF*(*T*) *P*_*initial*_ or *P*_*final*_ = *aG*(*S*)*P*_*initial*_). Here, large changes in AIC (presented as ΔAIC) with removal of temperature or salinity suggest the importance of the respective environmental component for reproduction and/or survival. Although there is no standard cut-off value for AIC, like there is for *p*-values, we used a conservative value of ΔAIC = 7, which corresponds to significance levels in the vicinity of 0.003 for nested models [41].

### Effects of Feeding Frequency on Survivorship and Asexual Reproduction

In order to determine the effect of feeding frequency on survivorship and asexual reproduction, polyps were exposed to five feeding regimes over a six-week period while held at the original culture conditions of 28°C ± 0.5°C and 33‰ ± 0.5‰. The five feeding regimes consisted of feeding every 1, 3, 7, or 14 days and a no food treatment over a six week period. Each feeding event consisted of food saturation for 24 hours (approximately one *Artemia* sp. nauplii per polyp per feeding event as outlined above) followed by a 100% water exchange 24 hours post feeding event with artificial pre acclimated sea water of 28°C ± 0.5°C and 33‰ ± 0.5‰. In the 14-day and no food treatments, water exchanges were conducted every seven days during the six-week experiment. Each replicate was photographed and the number of polyps in each replicate was determined at the start and in seven-day intervals during the experiment. Polyp density was analysed with a generalised linear mixed model with feeding frequency, time and the interaction between time and feeding frequency as fixed effects and replicate number as a random effect. This was analysed via proc glimmix in SAS, version 9.4 [42], using the ar(1) covariance matrix to describe temporal autocorrelation, and we assumed polyp density followed a negative binomial distribution with a log-link function, typical for count data [43]. We evaluated statistical inference tests with Wald F tests conducted in proc glimmix. The raw data file has been included in Supporting Information (S2 Dataset).

### Environmental Cues for Medusae Production

To explore feeding frequency, temperature, salinity and combinations thereof as potential cues for medusae production, each of the previously described six-week experiments was also monitored for medusae production. Medusae production for this species is in the form of monodisc strobilation [22] and each polyp was monitored for a change in body shape, formation of statoliths, attached and free swimming medusae. At the end of the six-week experiments each replicate of each treatment (80 temperature and salinity treatments and five feeding frequency treatments) was rapidly returned (shocked) back to the original culture condition of 28°C ± 0.5°C and 33‰ ± 0.5‰ and were monitored for a further four weeks (i.e., six weeks in treatment and four weeks post treatment). During this additional four-week period, the polyps were fed once per week followed by a complete water exchange 24 hours post feeding event.

## Results

### Thermal and Osmotic Effects on Survivorship and Asexual Reproduction

**Fig 1:**
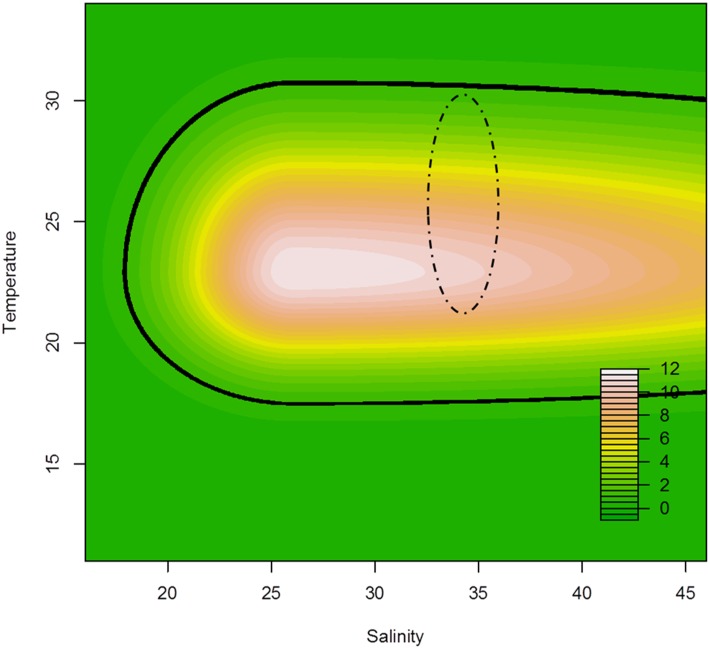
Maximum likelihood estimated proportional change in polyp density after six weeks plotted as a function of temperature and salinity from best-fit model. Model output describes proportional change in polyp density for different temperature and salinity values as described in Eq 1. Solid black line represents values of 1, where polyp density is expected to remain constant. The area encompassed by the dashed line indicates sea surface temperature and salinity levels that commonly occur within the waters of the Great Barrier Reef [44,45]. Models were fit to data collected from a matrix of eight temperature and ten salinity treatments, each replicated six times. Summary statistics describing the data used to generate the model are provided in Supporting Information (S1 Table).

Polyp survivorship and asexual reproduction rates significantly decreased as environmental values moved away from the optimal temperature and salinity, and removal of parameters describing the effects of each environmental variable suggested each variable was important (effect of removing temperature parameters by setting *F*(*T*) = 1: ΔAIC = 594, and effect of removing salinity parameters by setting *G*(*S*) = 1: ΔAIC = 164). These ΔAIC values are distinctly higher than our conservative baseline of ΔAIC = 7, and suggest that temperature and salinity alone are not sufficient to explain the data, and polyp survival and/or reproduction is indeed a function of both temperature and salinity. Summary statistics describing the data used to generate the model are provided in Supporting Information (S1 Table). The optimal temperature and salinity for polyp proliferation occurred at 22.9°C and 26.0‰, respectively, where the population increased by over 10 times during the six-weeks (see Table 1) compared to 100% mortality associated with both high and low temperatures and very low salinity levels. The temperature and salinity range that allowed for population stability (i.e., equal to or greater than 100% of the starting population) consisted of thermal treatments between 18°C and 31°C at salinity levels between 19‰ and 46‰. Although asexual reproduction was high at temperatures that commonly occur in the waters of the Great Barrier Reef, this was not the case with salinity, where the highest polyp proliferation was not encompassed by salinity levels that occur in the waters of the Great Barrier Reef (see Fig 1). While variation in population growth surrounding the optimum temperature and salinity was relatively symmetrical with respect to temperature (σ_*tl*_ = 3.47, σ_*th*_ = 5.01), population reproduction showed a steeper decrease with lower salinity (σ_*sl*_ = 5.19) than higher salinity (σ_*sh*_ = 31.07).

**Table 1:** Maximum Likelihood Estimates for Parameters Describing Proportional Change in Polyp Density of *Carukia barnesi*, After Six Weeks, Modelled as a Function of Temperature and Salinity.

Symbol	Description	Value	2.5% CL	97.5% CL
*a*	Maximum proportional change	11.48	9.00	14.95
σ_*tl*_	Temperature curve low	3.47°C	2.91°C	4.02°C
σ_*th*_	Temperature curve high	5.01°C	4.39°C	5.72°C
T_opt_	Optimum Temperature	22.91°C	21.92°C	23.86°C
σ_*sl*_	Salinity curve low	5.19‰	4.39‰	6.22‰
σ_*sh*_	Salinity curve high	31.07‰	21.70‰	89.95‰
S_opt_	Optimum Salinity	26.04‰	24.54‰	27.85‰

Model output describes proportional change in polyp density for different temperature and salinity values as described in Eqs 1, 2 and 3. Models were fit to data collected from a matrix of eight temperature and ten salinity treatments, each replicated six times. Estimated parameter values are based on the best fit parameters and 95% maximum likelihood profile confidence limits are provided.

### Effects of Feeding Frequency on Survivorship and Asexual Reproduction

**Fig 2:**
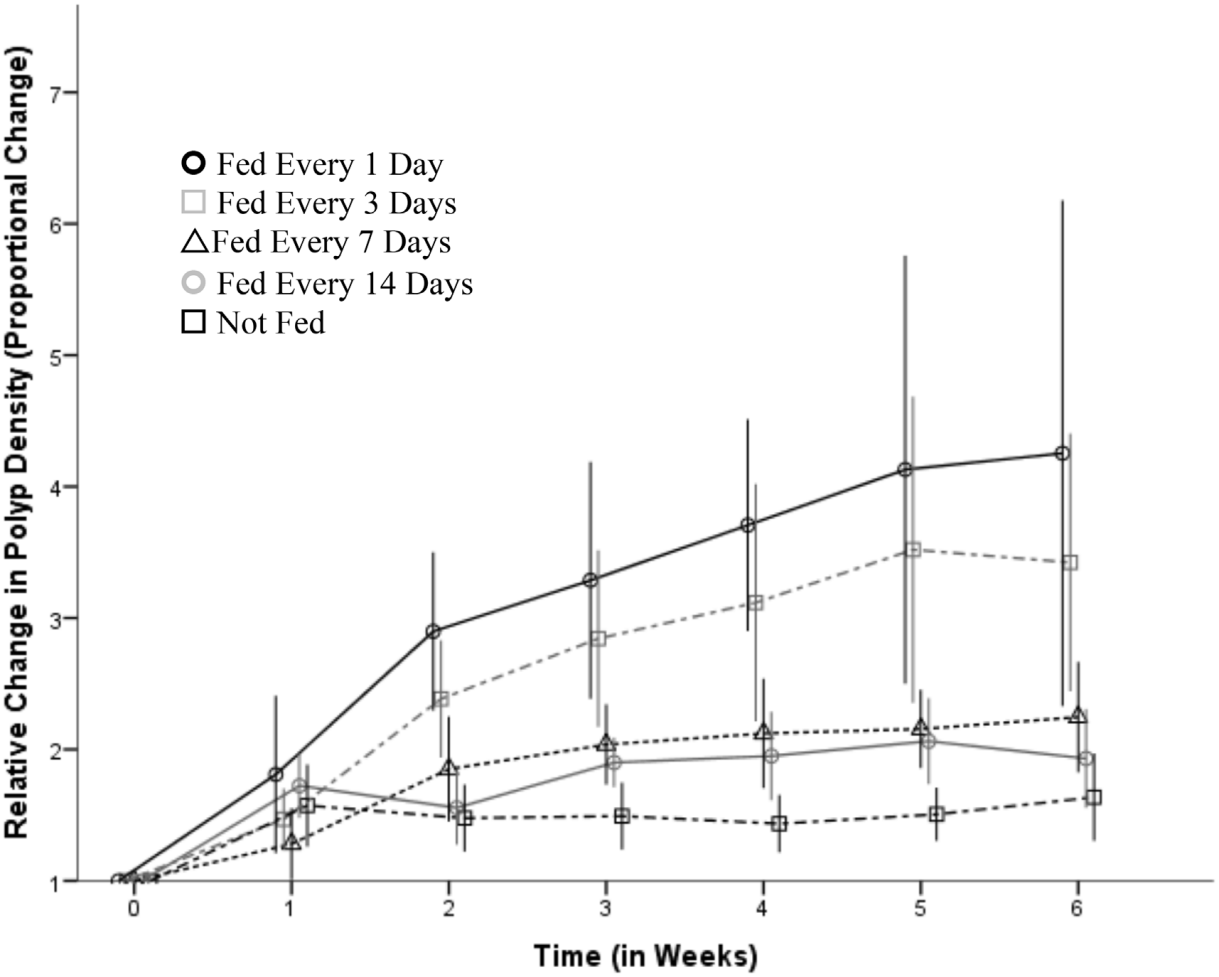
The relative change in *Carukia barnesi* polyp density, over six weeks of exposure, to five feeding regimes. The feeding levels consisted of: fed every day (dark circles); fed every three days (light squares); fed every seven days (dark triangles); fed every 14 days (light circles); unfed for 42 days (dark squares). Each of the five feeding treatments consisted of six independent replicates, each of which consisted of a mean staring polyp density of 20.5 polyps per well (*n* = 30, $\bar{x}$ = 20.5, *SD* = 2.8). The values for each time sequence were calculated as the proportional change in polyp density from the starting population density at time zero, where values of one indicate no population change and values above one indicate polyp proliferation through asexual reproduction. Values are reported as means and error bars represent 95% CI assuming a normal distribution. All counts were conducted in seven-day intervals however the values have been graphed offset on the time axis for clarity.

Feeding frequency significantly increased asexual reproduction (*F*_4, 25_ = 16.76, *p* < 0.001). In addition, there was significant temporal variation in polyp proliferation (*F*_6, 149_ = 78.65, *p* < 0.001) and the positive effect of feeding frequency became incrementally stronger over time (*F*_24, 149_ = 5.21, *p* < 0.001). Polyps that were fed daily increased mean population numbers by over three times during the six-weeks, and polyps that were unfed increased in population by 0.5 times over the same time frame (Fig 2).

### Environmental Cues for Medusae Production

Temperature, salinity, and feeding frequency, and variations thereof, did not trigger medusae production during the six-week experiments or during the four-week post treatment monitoring period. Only one polyp went through medusae production during the ten-weeks. This occurred in the 31°C and 46‰ treatment and was identified during week five of the experiment. As a percentage, one polyp undergoing medusae production during this experiment was not significant. Returning the polyps to the original culture conditions of 28°C ± 0.5°C and 33‰ also did not cause medusae production over the four-week post experiment monitoring period.

## Discussion

*Carukia barnesi* polyps had a high tolerance to osmotic treatments with 100% survivorship from treatments between 18 and 46‰ (Fig 1), which indicates that this species may inhabit areas with a high degree of osmotic variation. The osmotic treatment that yielded the highest degree of polyp proliferation was 26‰. Salinity levels this low do not occur in the waters of the Great Barrier Reef or in the Coral Sea, however these low salinity conditions are a common occurrence in estuarine systems [46–49]. These results indicate that although the polyp stage does not seem restricted to low salinity waters, the observed increase in asexual reproduction under low salinity conditions suggests that the polyp stage is probably an estuarine inhabitant. Cubozoans that have coastal or estuarine polyp stages have previously been reported for two species; *C*. *fleckeri* polyps were located *in situ* attached to the underside of rocks near a river mouth in Queensland [4], and polyps of *C*. *marsupialis* were once located attached to bivalve shells in a mangrove habitat in Puerto Rico [12]. To date, these are the only two discoveries of cubozoan polyps *in situ* globally, however at least one other Australian cubozoan polyp, those of *C*. *bronzie*, has also been suggested to be estuarine [2]. The possibility that *C*. *barnesi* has an estuarine polyp stage has not previously been considered primarily due to their distinctly oceanic medusa stage [5,6,34] compared to species which are known to be primarily coastal, such as *C*. *fleckeri* and *C*. *bronzie* [2,3,50–52]. There have been no reported sightings, or documented stings caused by *C*. *barnesi* from estuaries; however this does not discount the possible presence of medusae in these areas when they are newly detached and small. It is currently unknown how the polyps enter low salinity habitats and nothing is known about the osmotic tolerance of the medusa stage or where or when the medusae spawn. In speculation, due to there being no reports of adult *C*. *barnesi* medusae within estuarine systems, it is plausible that the eggs, that are known to have a long encapsulated planula stage from six days to six months [22], are transported inshore on currents. Future research is required on the medusa stage, such as determining their osmotic tolerance, to better understand how the life cycle is completed *in situ*.

Temperature also affected both the survivorship and asexual reproduction rate of *C*. *barnesi* polyps. There was notable symmetry between the high and low temperature curves with a modelled optimum temperature of 22.9°C (Table 1). As the thermal treatments moved away from optimum the amount of polyp proliferation through asexual reproduction was reduced. The minimum and maximum thermal constraints of the polyps were between 18°C and 31°C, which indicates that the polyp stage has a suitable operating envelope of approximately 13 degrees, whereby positive population growth is possible over a period of six weeks.

The southern distribution limit of the medusa stage of *C*. *barnesi* is not well established. However, at present it is considered to be near Fraser Island based on sting and capture records (Seymour unpublished). The winter (July) sea surface temperature near Fraser Island typically averages 20°C and increases to 27°C during the summer (February) [45], which is within the thermal capacity range for population growth of the polyp stage and encompasses their thermal optimum of 22.9°C. Although the temperature profile of *C*. *barnesi* polyps seems to fit well with the water temperature near Fraser Island, it is expected that the water temperature in estuarine systems near river mouths, which are predominately large river systems along the eastern coast of Queensland, to be significantly lower in temperature than the adjacent open ocean. For example, temperatures near the mouth of the Noosa Rivers (approximately 100km south of Fraser Island) are known to average as low as 18°C during the winter [53] compared to 20°C in the open ocean near Fraser Island [45]. Therefore, the polyp stage of *C*. *barnesi* may be restricted to warmer estuarine areas where the winter water temperature does not drop as low as 18°C north of Fraser Island. This suggests that the medusa stage may limit the southern distribution of this species, though no data exists on the thermal tolerance of the medusa stage to confirm this.

The observed rate of asexual reproduction of *C*. *barnesi* during these experiments was high compared to another cubozoan polyp, *A*. nr *mordens* [26]. For example, a maximum polyp population increase of 50% was recorded for *A*. nr *mordens* over six weeks [26] compared to over 1000% in *C*. *barnesi* during this replicated experiment (Fig 1). This high rate of reproduction is expected to increase the number of polyps able to produce medusae at any one time and in turn influence the abundance of medusae present. Highly reproductive populations are expected to make use of rapid environmental changes [54–56]. Purely in speculation, and assuming these polyps are estuarine and require hard substrate for attachment similar to other cnidarian polyps [57–60], increasing manmade structures within marine environments (e.g., marinas, boat ramps and mooring platforms), may increase the available hard substrate for polyp attachment. This anthropogenic effect has been suggested to positively impact on scyphozoan polyp populations [61–65]. Not surprisingly, increased asexual reproduction was correlated with increased feeding frequency. Therefore, changes in estuarine trophic dynamics, primarily eutrophication, may lead to increased copepod density [66,67], presumably a primary food source for polyps and early stage medusae, which may influence polyp proliferation and in turn affect medusae abundance.

Some cubozoan polyps are known to use environmental factors, such as temperature [19,23–25], or reduced food availability [26], as a cue for medusae production. However, temperature, salinity, feeding frequency, and combinations of these factors, did not trigger medusae production in *C*. *barnesi*. This suggests that the polyps may use a different cue for synchronous medusae production. Because of the consistency of the first arrival of the medusae stage over the last 50 years [68], it is possible that this species uses increasing photoperiod as a cue due to the interannual variation in environmental parameters such as temperature and salinity. This type of cue has also been suggested for *C*. *fleckeri* due to the consistency of occurrence during a seven-year study [3]. There is also evidence that the stinger season length has increased over the last 50 years from 15 days to 151, which has been speculated to be caused by increasing global sea temperatures [68]. Therefore it is possible that *C*. *barnesi* polyps use photoperiod to initiate synchronous medusae production but continue to produce medusae throughout the season until the winter water temperature becomes too low.

Future research should pursue determining the thermal and osmotic parameters of the medusa stage of *C*. *barnesi*, and experimentally determine possible environmental cues for medusae production. Understanding the contributing factors that lead the spatial and temporal variability in medusae abundance may in turn prove valuable to predicting future *C*. *barnesi* abundance, and/or distributional range extensions, under projected sea temperature rise scenarios.

## Supporting Information

S1 Dataset *Carukia barnesi* Polyp Count Data Associated With “Thermal and Osmotic Effects on Survivorship and Asexual Reproduction”. Variable list/description: Sample identification number; Well number; Treatment number; Temperature (°C); Salinity (‰); Time (weeks) (e.g., Time 0 indicates start of experiment and Time 6 indicates after 6 weeks; Total (total number of polyps counted); Proportion (proportional change in polyp density), this variable was calculated as Total polyps at Time 6/Total polyps at Time 0.
(CSV)

S2 Dataset *Carukia barnesi* Polyp Count Data Associated With “Effects of Feeding Frequency on Survivorship and Asexual Reproduction”. Variable list/description: Sample identification number; Time (weeks) (e.g., Time 0 indicates start of experiment and Times 1–6 indicate after 1–6 weeks; Well number; Temperature (°C); Salinity (‰);Total (total number of polyps counted); Proportion (proportional change in polyp density), this variable was calculated as Total polyps at Times 1–6/Total polyps at Time 0.
(CSV)

S1 R Code The R Code Used for Analysis of “Thermal and Osmotic Effects on Survivorship and Asexual Reproduction”. (TXT)

S1 Table Mean Values and Standard Errors of the Number of Polyps Present after Six Weeks Exposure to a Matrix of Eight Temperatures and Ten Salinity Treatments. All values were calculated as the relative change in polyp density and are presented as proportional change, where values above one indicate population increase through asexual reproduction and values below one indicate polyp mortality. There were six independent replicates for each treatment, therefore *n* = 6 for all means presented below and standard errors were calculated assuming a normal distribution.
(RTF)
