# Supplementary material for: Thermal and Osmotic Tolerance of ‘Irukandji’ Polyps: Cubozoa; Carukia barnesi
Source: PLoS One. 2016 Jul 21;11(7):e0159380. doi: 10.1371/journal.pone.0159380 (PMC4956319; doi:10.1371/journal.pone.0159380)
Supplement: S1 Table — All values were calculated as the relative change in polyp density and are presented as proportional change, where values above one indicate population increase through asexual reproduction and values below one indicate polyp mortality. There were six independent replicates for each treatment, therefore n = 6 for all means presented below and standard errors were calculated assuming a normal distribution. (RTF) [file pone.0159380.s004.rtf]

	11ºC	14ºC	18ºC	21ºC	25ºC	28ºC	31ºC	34ºC	
16‰	 x̅ = 0.00
SE = 0.00	 x̅ = 0.00
SE = 0.00	 x̅ = 0.00
SE = 0.00	 x̅ = 0.20
SE = 0.11	 x̅ = 0.76
SE = 0.11	 x̅ = 0.01
SE = 0.01	 x̅ = 0.01
SE = 0.01	 x̅ = 0.00
SE = 0.00	
19‰	x̅ = 0.00
SE = 0.00	x̅ = 0.00
SE = 0.00	x̅ = 0.00
SE = 0.00	x̅ = 0.45
SE = 0.11	x̅ = 1.36
SE = 0.17	x̅ = 1.25
SE = 0.09	x̅ = 0.00
SE = 0.00	x̅ = 0.00
SE = 0.00	
22.5‰	x̅ = 0.00
SE = 0.00	x̅ = 0.00
SE = 0.00	x̅ = 1.65
SE = 0.22	x̅ = 3.66
SE = 0.38	x̅ = 8.96
SE = 1.32	x̅ = 1.85
SE = 0.21	x̅ = 1.56
SE = 0.43	x̅ = 0.00
SE = 0.00	
26‰	x̅ = 0.00
SE = 0.00	x̅ = 0.00
SE = 0.00	x̅ = 1.76
SE = 0.19	x̅ = 3.78
SE = 1.06	x̅ = 7.29
SE = 0.96	x̅ = 1.84
SE = 0.19	x̅ = 2.17
SE = 0.12	x̅ = 0.00
SE = 0.00	
29‰	x̅ = 0.00
SE = 0.00	x̅ = 0.00
SE = 0.00	x̅ = 2.86
SE = 0.24	x̅ = 4.19
SE = 0.34	x̅ = 3.05
SE = 1.01	x̅ = 1.85
SE = 0.20	x̅ = 1.83
SE = 0.29	x̅ = 0.00
SE = 0.00	
33‰	x̅ = 0.00
SE = 0.00	x̅ = 0.00
SE = 0.00	x̅ = 3.14
SE = 0.50	x̅ = 4.17
SE = 0.86	x̅ = 3.13
SE = 1.00	x̅ = 2.25
SE = 0.16	x̅ = 2.03
SE = 0.40	x̅ = 0.00
SE = 0.00	
36‰	x̅ = 0.00
SE = 0.00	x̅ = 0.00
SE = 0.00	x̅ = 3.09
SE = 0.37	x̅ = 2.26
SE = 0.61	x̅ = 4.92
SE = 0.31	x̅ = 1.84
SE = 0.14	x̅ = 1.77
SE = 0.20	x̅ = 0.00
SE = 0.00	
39‰	x̅ = 0.00
SE = 0.00	x̅ = 0.00
SE = 0.00	x̅ = 2.94
SE = 0.28	x̅ = 3.48
SE = 0.52	x̅ = 4.63
SE = 1.09	x̅ = 1.76
SE = 0.21	x̅ = 1.50
SE = 0.24	x̅ = 0.00
SE = 0.00	
42.5‰	x̅ = 0.00
SE = 0.00	x̅ = 0.00
SE = 0.00	x̅ = 1.66
SE = 0.42	x̅ = 4.84
SE = 0.74	x̅ = 3.76
SE = 0.95	x̅ = 1.94
SE = 0.10	x̅ = 1.65
SE = 0.19	x̅ = 0.00
SE = 0.00	
46‰	x̅ = 0.00
SE = 0.00	x̅ = 0.00
SE = 0.00	x̅ = 2.16
SE = 0.35	x̅ = 2.00
SE = 0.40	x̅ = 3.14
SE = 0.27	x̅ = 1.72
SE = 0.11	x̅ = 1.03
SE = 0.23	x̅ = 0.00
SE = 0.00	
